# Cigarette smoking patterns in relation to religiosity and familial support among Iranian university students: A Latent Class Analysis

**DOI:** 10.18332/tid/92649

**Published:** 2018-07-10

**Authors:** Sima Afrashteh, Haleh Ghaem, Ali Gholami, Hamid Reza Tabatabaee, Abbas Abbasi-Ghahramanloo

**Affiliations:** 1 Bushehr University of Medical Sciences, Bushehr, Iran; 2 Student Research Committee, Shiraz University of Medical Sciences, Shiraz, Iran; 3 Research Center for Health Sciences, Institute of Health, Department of Epidemiology, School of Health, Shiraz University of Medical Sciences, Shiraz, Iran; 4 Department of Public Health, School of Public Health, Neyshabur University of Medical Sciences, Neyshabur, Iran; 5 Department of Epidemiology, Faculty of Health, Iran University of Medical Sciences, Tehran, Iran; 6 Health Management Research Center, Baqiyatallah University of Medical Sciences, Tehran, Iran

**Keywords:** latent class analysis, cigarette smoking, students, familial support, religiosity

## Abstract

**INTRODUCTION:**

Entering University is an important developmental milestone that might be associated with cigarette smoking. The aim of this study was to identify the subgroups of university students on the basis of cigarette smoking patterns, and to assess the role of familial support and religious beliefs on membership in specific subgroups.

**METHODS:**

This cross-sectional study was performed in 2016 using multistage random sampling among students of Bushehr University (n=977). Anonymous, structured questionnaires were distributed to the students in each selected class. Cigarette smoking prevalence was assessed in three time intervals: lifetime, last year, and last month. All of the analyses were performed using PROC LCA in the SAS software.

**RESULTS:**

The lifetime, last-year and last-month prevalence of cigarette smoking was 13.7%, 10.0% and 7.0%, respectively. In this study, the prevalence of passive smoking was relatively high (15.3%) among students. Four latent classes were identified: 1) non-smoker 58.2%, 2) passive smoker 31.3%, 3) moderate smoker 3.4%, and 4) heavy smoker 7.1%. The prevalence of cigarette smoking of close friends was: 73% among passive smokers, 81% for heavy smokers and 63% for moderate smokers. Being male (OR=4.42, 95% CI; 2.90–6.74) and a higher score of religious beliefs (OR=0.97, p<0.001 95% CI; 0.96–0.98) were associated with the heavy smoker class.

**CONCLUSIONS:**

Among students at Bushehr University in Iran, 10.5% were either moderate or heavy smokers in 2016. These results point out the critical importance of designing specific preventive interventional programs for university students. Higher level of religiosity may serve as a preventive factor in engaging in cigarette smoking.

## INTRODUCTION

According to the World Health Organization (WHO) report, tobacco consumption is one of the biggest global public health threats, accounting for more than 7 million deaths each year^1^. More than 6 million of the deaths are the result of direct tobacco use while around 890000 are the result of non-smokers being exposed to second-hand smoke^1^. About 80% of the world’s smokers (more than 1 billion) live in low-and middle-income countries^1^.

Initiation of tobacco use among youth who are facing university entrance is commonly due to new types of social and emotional issues. Tobacco use is associated with adverse health-related consequences among young individuals^2^.

The prevalence of cigarette smoking varies widely amongst university students in developing countries, with the prevalence of cigarette smoking being 60.2% in Bangladesh, 22.2% in Saudi Arabia, 30% in Palestine, 26.7% in India and 20.7% in Syria^3-7^. Results from a meta-analysis among Iranian university students reported the highest and lowest rates of cigarette smoking as 39.9% and 13.4%, respectively^8^. Allahverdipour et al.^9^ showed that among nine universities of Tabriz (North West of Iran), 15.8% of students were cigarette smokers. Findings from the Monitoring The Future (MTF) and the European School survey Project on Alcohol and other Drugs (ESPAD) studies showed that the prevalence of cigarette smoking in the past 30 days was 11.3% and 21% in American and European university students, respectively^10,11^.

There are differences in the definition of being a smoker in the literature. For this reason, it is not possible to compare the prevalence rates. Large and well documented studies (like MTF) used three time intervals (life time, last year, and last month) in reporting cigarette smoking prevalence. To the best of our knowledge, there is no study that reported the cigarette smoking prevalence among university students in Iran in the above mentioned three time intervals.

The findings of the previous studies suggested that some factors such as parental smoking, peer smoking and low self-confidence increase the risk of cigarette smoking initiation among university students in Iran and other countries, while religion and familial support are protective factors against smoking^9,12-14^. Less is known, however, about the effect of religion and familial support in different stages of smoking among university students.

Conducting additional studies on cigarette smoking among university students is necessary as the cigarette smoking rate among this group can be a convenient indicator of risk taking behavior such as cigarette smoking among young people^15^.

Latent Class Analysis (LCA) is an effective statistical tool to identify subgroups of the target population^16^. A study conducted in the US using the LCA technique identified four latent subclasses of cigarette smoking: puffer, social smoker, moderate smoker, and heavy smoker^17^. This study aims to investigate student subgroups in an Iranian University, according to cigarette smoking patterns using LCA technique, and to determine the prevalence and correlates of membership in each class.

## METHODS

This cross-sectional study was performed on 977 students in Bushehr, south of Iran in 2016. The sample was selected through multi-stage sampling. At the first stage, total numbers of students were calculated. At the second stage, in proportion to the number of students in each faculty, the sample size was calculated from each faculty. Finally, a number of classes were randomly selected from each faculty. All students of the selected classes were recruited. Data were collected using a self-administered questionnaire. All participants were given 15–20 minutes to voluntarily complete the questionnaires.

This self-administered questionnaire comprised four parts. First part assessed demographic information such as age, gender, etc. The other parts evaluated information regarding various substance use from hookah, cigarette smoking and illicit drug use. The validity and reliability of this questionnaire were measured in previous studies^18,19^.

The questionnaire collected information on cigarette smoking status, including lifetime, past year, and past 30-day smoking, in addition to parental and peer smoking over the past 30 days. The prevalence of smoking in the three time intervals was assessed by the survey question: ‘Do you smoked cigarette ever, over the past year, and over the past 30 days?’. The prevalence of smoking among family members and friends was assessed by asking: ‘Have any of your friends or family members smoked over the past 30 days?’.

Familial support was measured using Aneshensel and Sucoff’s 13-item questionnaire. Some examples of the scale phrases were as follow: ‘my mom or dad make me trust them’, and ‘they truly understand me’. The scale items were rated on a scale 1 to 5 covering the responses: completely agree, agree, disagree, completely disagree, and no idea. Cronbach’s alpha^20^ was computed at 0.86.

Additionally, the 28-item Kendler’s general religiosity scale translated into Persian by Farhadinasab and colleagues^20^ was used to measure the general religiosity of the students. Some examples of the scale phrases were: ‘I always think of God’, ‘I sense God’s direct and indirect attention through me’, and ‘I see God’s signs in my life everyday’. The 5-point items were rated on a scale 1 to 5 with responses: completely agree, agree, neither agree nor disagree, disagree, and completely disagree. Cronbach’s alpha was computed at 0.97.

The statistical analysis was performed using the LCA technique. According to this model, a number of observable variables are aggregated to represent a categorical latent variable. The G2 (The likelihood ratio test statistic) is calculated to select the best fitting model. Also, the Akaike Information Criterion (AIC) and Bayesian Information Criterion (BIC) were applied to select the best fitted model. The lowest rate of G2, AIC and BIC indicate the best fitting model. Six dichotomous observable variables (i.e. indicators) were selected for the subgrouping of students. These indicators were lifetime smoking, last-year smoking, last-month smoking, passive smoking, cigarette smoking of family members, and cigarette smoking of close friends. After finalizing the model, religious beliefs, familial support as well as age, gender, marital status and living place were entered as covariates in the model. All statistical analyses were performed using PROC LCA in the SAS 9.2 software.

## RESULTS

This study involved 977 students aged 17–39 years, of which 41.4 % were males. As shown in Table 1, the lifetime, last-year and last-month rates of cigarette smoking were 13.7%, 10%, and 7%, respectively. Moreover, 15.3% of the students were exposed to cigarette smoking (passive smoking) over the past 7 days. The findings also revealed that the rates of smoking in the last month among family members and friends were 25.7% and 31%, respectively.

**Table 1:** Cigarette smoking status by ‘familial support’ and ‘religious beliefs’ in a sample of Iranian students in Bushehr

*Items*	*Score of familial support*	*p*	*Score of religious beliefs*	*p*	*Total n=977*

	*Mean ± SD*		*Mean ± SD*		*%*
**Life time smoking**					
Yes	46.02 ± 11.63	<0.001	96.98 ± 25.29	<0.001	13.7
No	51.29 ± 9.94		115.05 ± 18.48		86.3
**Last year smoking**					
Yes	46.10 ± 12.57	<0.001	95.04 ± 26.68	<0.001	10.0
No	51.07 ± 9.95		114.52 ± 18.72		90.0
**Last month smoking**					
Yes	45.27 ± 13.27	<0.001	97.40 ± 26.00	<0.001	7.0
No	50.96 ± 9.99		113.70 ± 19.59		93.0
**Passive smoking (one day or more in the past week)**					
Yes	46.97 ± 10.94	<0.001	102.78 ± 23.50	<0.001	15.3
No	51.22 ± 10.10		114.33 ± 19.41		84.7
**Cigarette smoking of family members (last month)**					
Yes	49.62 ± 10.51	0.094	107.02 ± 22.13	<0.001	25.7
No	50.89 ± 10.27		114.51 ± 19.54		74.3
**Cigarette smoking of close friends (last month)**					
Yes	48.31 ± 10.82	<0.001	106.06 ± 22.60	<0.001	31.0
No	51.58 ± 9.97		115.51 ± 18.77		69.0

Table 1 also presents the conditional distribution of score of familial support and religious beliefs at each level of the cigarette smoking status. Cigarette smoking of family members had no significant relationship with the score of familial support. Lifetime, last-year and last-month cigarette smoking, passive smoking, cigarette smoking of family members and cigarette smoking of close friends had a significant relationship with the score of religious beliefs. Active smokers in all three time intervals and passive smokers have lower scores of religious beliefs and familial support than non-smokers. Students whose family members were smokers had low scores of religious beliefs. Also, students whose friends were cigarette smokers had low scores of religious beliefs and familial support.

We used six binary variables to conduct the LCA analysis. We attempted to fit the LCA models with classes ranging from 1 to 8 (Table 2). Different measures of model assessment are shown in Table 2. The 4-class model had the lowest value of AIC and the 3-class model had the lowest value of BIC. Finally, the 4-class model was chosen as the best fit for the data based on the interpretability and plausibility of the results.

**Table 2:** Comparison of LCA Models with different latent classes based on model selection statistics

*Number of latent class*	*Number of parameters estimated*	*G^2^*	*df*	*AIC*	*BIC*	*Maximum log-likelihood*	*p*
1	6	1179.45	57	1191.45	1220.76	-2534.50	< 0.00001
2	13	180.37	50	206.37	269.87	-2034.96	< 0.00001
3	20	56.56	43	96.56	194.25	-1973.06	0.080454
4	27	29.87	36	83.87	215.76	-1959.71	0.754342
5	34	18.93	29	86.93	253.00	-1954.24	0.92307
6	41	12.70	22	94.70	294.96	-1951.12	0.941192
7	48	9.67	15	105.67	340.12	-1949.61	0.839968
8	55	7.29	8	117.29	385.93	-1948.42	0.505691

LCA: latent class analysis, AIC: Akaike information criterion, BIC: Bayesian information criterion.

The results of the four LCA classes model showed that differences between the expected and observed frequency of response patterns were not statistically significant (G2=29.87, df=36, p=0.75). After finalizing the model, age, gender, marital status, living place, religious beliefs and familial support were entered as covariates to the model. The first row of Table 3 shows the probability of membership in each latent class. The first class, *non-smoker*, described 58.2% of the students, while the second class, *passive smoker*, third class, *moderate smoker*, and fourth class, *heavy smoker*, described 31.3%, 3.4% and 7.1% of the students, respectively.

**Table 3:** The four Latent Classes Model of cigarette smoking and related covariates

	*Latent class*			

	*Non-smoker*	*Passive smoker*	*Moderate smoker*	*Heavy smoker*
**Latent class prevalence**	0.582	0.313	0.034	0.071
**Item-response probabilities**	Probability of a ‘Yes’			
**life time smoking**	0.018	0.071	**0.993**	**0.997**
**last year smoking**	0.000	0.000	**0.871**	**0.994**
**last month smoking**	0.003	0.008	0.145	**0.852**
**passive smoking**	0.037	0.241	0.015	0.775
**cigarette smoking of family members (last month)**	0.122	0.445	0.446	0.443
**cigarette smoking of close friends (last month)**	0.007	**0.726**	**0.627**	**0.812**

The probability of a ‘No’ response can be calculated by subtracting the item-response probabilities shown above from 1.

* Item-response probabilities >0.5 in bold to facilitate interpretation.

As shown in the second section of Table 3, the probability of cigarette smoking among family members and friends was higher in the *heavy smoker* and *passive smoker* classes than the *non-smoker* class. Similarly, the probabilities of smoking in the lifetime, last year and last month were higher in the *heavy smoker* class than *non-smoker* and *passive smoker* classes. In the *moderate smoker* class the probability of smoking in the lifetime and last year was high but in the last month was low.

The odds ratio associated with all covariates for all class memberships are shown in the last section of Table 4. Higher scores of religious beliefs decreased the odds of membership in *passive smoker* (OR=0.989, 95% CI: 0.98–0.99), *moderate smoker* (OR=0.966, 95% CI: 0.95–0.97) and *heavy smoker* classes (OR=0.971, 95% CI: 0.96–0.98) compared to the non-smoker class. Being male increases the odds of membership in the *passive smoker* (OR=2.80, 95% CI: 2.22–3.53), and *heavy smoker* classes (OR=4.42, 95% CI: 2.90–6.74) compared to the non-smoker class.

**Table 4:** Predictors of membership in latent classes of cigarette smoking

*Predictors*	*p*	*Non-smoker*	*Passive smoker*	*Moderate smoker*	*Heavy smoker*

		*OR (95%CI)*	*OR (95%CI)*	*OR (95%CI)*	*OR (95%CI)*
**Age**	0.729	Reference	1.043 (0.99–1.09)	1.068 (0.95–1.18)	1.029 (0.95–1.11)
**Gender (being male)**	<0.001	Reference	2.800 (2.22–3.53)	1.397 (0.80–2.42)	4.423 (2.90–6.74)
**Marital status (being single)**	0.963	Reference	0.977 (0.69–1.38)	1.220 (0.51–2.89)	0.878 (0.46–1.65)
**Living in single house**	0.147	Reference	0.720 (0.47–1.09)	0.805 (0.30–2.14)	1.580 (0.90–2.74)

The effect of familial support was not significant in the prediction of membership in the various classes.

## DISCUSSION

In the present study, the lifetime, past-year and the past-month rates of smoking are 13.7%, 10%, and 7%, respectively. In comparison with American, European, Indian and Chinese university students, the prevalence of smoking is quite low in Iranian students. The prevalence results of smoking in this study are similar to those of previous studies for Iran. The differences between Iranian and the other students may arise from different cultural and social norms, and from different definitions of being a smoker.

In the present study, we evaluated cigarette smoking patterns and were able to detect four distinct classes: non-smoker, passive smoker, moderate smoker, and heavy smoker.

The results suggest that being male is associated with increased odds of membership in the *passive smoker* and *heavy smoker* classes. This finding is supported by similar studies from Iran^16,21^.

The findings of the present study also show that higher religious belief scores decreased the odds of membership in the *passive smoker, moderate smoker,* and *heavy smoker* classes, compared to the non-smoker class. The results of the present study are consistent with other studies that emphasized the role of religiosity in the prevention of risk-taking behaviors, especially cigarette smoking. Mohammadpoorasl et al.^16^ in a similar study among students found that religious beliefs decreased the odds of membership in the sexual-risk taker class and high risk class compared to low risk class. Studies have shown that religious beliefs are associated with positive mental health outcomes leading to mental relaxation and pleasant living^3,9,20,22^. The fact that we belong to God may reduce the potential negative consequences when facing problems. This attitude can strengthen non-smoking behaviour, and subsequently prevent smoking^22^.

Several implications for public health and policy makers can be drawn from this finding. Religious belief is a critical and influential factor in the prevention of the initiation of cigarette smoking. Religious belief could be used as a marker for risk along with other related factors for the identification and implementation of interventional programs for prevention of smoking.

For example, Bailey et al.^23^ in a prospective study concluded that interventions to increase smoking abstinence may be more effective if the participants draw on ties to religious organizations. However, this study showed that it is unlikely that religious involvement will affect smoking cessation effectiveness.

The results of the present study revealed the important role of cigarette smoking by close friends in classifying students. The results of a prospective study indicated that friends are the only significant predictors of substance use in early adulthood^24^. However, the role of family is quite critical in choosing friends.

Several aspects of this study can limit the application of the findings. First, the cross-sectional nature of the study serves to provide evidence for the relationship between independent variables and cigarette smoking status and does not establish causality. Second, generalization of the results is limited to the students in the city of Bushehr. Finally, confounding variables, such as psychiatric disorders were not accounted for in the analysis.

For future studies, longitudinal studies are required to determine and monitor the incidence rate of smoking rates and its correlates among students.

## CONCLUSIONS

In the present study, we evaluated the prevalence and patterns of cigarette smoking among students of Bushehr city. Results revealed that 10.5 % of students were either moderate or heavy smokers. The findings highlighted the protective impact of religious beliefs on the odds of membership in the different latent classes of smoking. Consequently, focusing on education about religious beliefs and emphasizing religious-based interventions can be considered as elements of effective preventative programs for university students.
